# Preliminary Studies on the Mechanism of Antifungal Activity of New Cationic β-Glucan Derivatives Obtained from Oats and Barley

**DOI:** 10.1021/acsomega.2c05311

**Published:** 2022-10-27

**Authors:** Kamil Kamiński, Katarzyna Hąc-Wydro, Magdalena Skóra, Małgorzata Tymecka, Magdalena Obłoza

**Affiliations:** † Faculty of Chemistry, Jagiellonian University, Gronostajowa 2 Street, 30-387Kraków, Poland; ‡ Department of Infections Control and Mycology, Chair of Microbiology, Jagiellonian University Medical College, Czysta 18 Street, 31-121Kraków, Poland

## Abstract

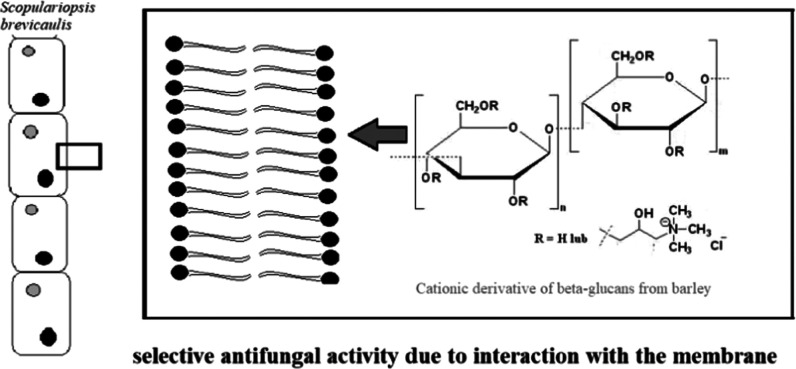

New chemical structures with antifungal properties are highly desirable from the point of view of modern pharmaceutical science, especially due to the increasingly widespread instances of drug resistance in the case of these diseases. One way to solve this problem is to use polymeric drugs, widely described as biocidal, positively charged macromolecules. In this work, we present the synthesis of new cationic β-glucan derivatives that show selective antifungal activity and at the same time low toxicity toward animal and human cells. Two β-glucans isolated from oats and barley and modified using glycidyltrimethylammonium chloride were obtained and evaluated for biocidal properties on the cells of mammals and pathogenic fungi and bacteria. These compounds were found to be nontoxic to fibroblast and bacterial cells but showed selective toxicity to certain species of filamentous fungi (*Scopulariopsis brevicaulis*) and yeasts (*Cryptococcus neoformans*). The most important aspect of this work is the attempt to explain the mechanisms of action of these compounds by studying their interaction with biological membranes. This was achieved by examining the interactions with model biological membranes representative of given families of microorganisms using Langmuir monolayers. The data obtained partly show correlations between the results for model systems and biological experiments and allow indicating that the selective antifungal activity of cationic β-glucans is related to their interaction with fungal biological membranes and partly lack of such interaction toward cells of other organisms. In addition, the obtained macromolecules were characterized by spectral methods (Fourier transform infrared (FTIR) and ^1^H nuclear magnetic resonance (NMR) spectroscopies) to confirm that the desired structure was obtained, and their degree of modification and molecular weights were determined.

## Introduction

The number of reported cases of fungal infection has increased dramatically over the last decade.^1^ This phenomenon is mainly related to the increasing populations of people predisposed to opportunistic mycoses, especially patients with impaired immune systems and other primary diseases like *diabetes mellitus*.^2^ The emergence of fungal infections is also associated with therapeutic difficulties, mainly the growing numbers of strains resistant to currently available antimycotics.^3^ The most worrying concern is the incidence of infections caused by multi-drug-resistant strains.^4−7^ The number of tools against fungal infections available to modern medicine is very limited. There are only four main families of chemical structures used to combat these diseases (azoles, polyenes, echinocandins, and pyrimidine analogues).^3^ This state of things means that antifungal drugs are most often used according to broad-spectrum antibiotic tactics^8^ without identifying the species attacking the patient. This approach is associated with much controversy among medical practitioners,^9^ and although lucrative for the pharmaceutical industry, which does not have to invest heavily in drug discovery,^10^ it is unacceptable in the long run. It would be advisable for the treatment of such diseases to focus on targeted therapy and the idea of narrow-spectrum antibiotics in the design of new families of active structures because of the phenomenon of drug resistance and the increasing number of patients.^11,12^ An interesting target for the biological activity of new antimycotic compounds is the fungal membrane,^13^ which is characterized by a high degree of diversity across species and at the same time is dramatically different in terms of composition from the membrane of bacteria and higher organisms. Such tactics in the design of antimicrobial drugs would make it possible to obtain highly selective compounds that do not affect other beneficial or pathogenic microorganisms and at the same time do not contribute to the emergence of drug resistance. There is a big potential here, in our opinion, for macromolecular drugs that, unable to significantly penetrate cells due to their large mass, will concentrate their activity on the barriers surrounding the cytoplasm (e.g., damaging its integrity^14^). There are reports in the literature that go so far as to postulate that the membrane is partly responsible for drug resistance and should therefore be the primary target for new antifungal drugs.^15^ The use of positively charged polymers in antifungal applications has been proposed postulatively in mainly agricultural applications^16^ and also to a small extent for medical use^17^ without clearly indicating a mechanism of action. Their biocidal use against bacteria^18^ and partly against higher cells^19^ has been reported in the literature, while the mechanisms responsible for these phenomena are also not clearly explained. This problem calls for research focusing on models specifically designed for fungi whose membranes differ from those of cells of other kingdoms. In this work, only such systems were tested for sensitivity to new cationic derivatives of β-glucans isolated from barley and oat wholemeal flour. β-glucans are polysaccharides widely reported in the literature with postulated health-promoting properties concerning a wide range of conditions related to digestion,^20,21^ immune system function,^21^ and blood cholesterol levels.^22^ The limiting aspect regarding this group of polymers is their moderately low water solubility, which can be improved by introducing charged functional groups into their structure. In this work, we propose the introduction of a quaternary amine (by modification with glycidyltrimethylammonium chloride), which we postulate will further broaden the already vast biological activity of these compounds and greatly improve solubility. In this paper, we describe results confirming the antifungal properties of such novel systems against selected pathogenic fungal species while lacking toxicity against mammalian fibroblasts and bacteria. Representative species of fungi responsible for various most common infections in humans were selected for the study (species of *Candida*, *Aspergillus*, *Trichophyton*, *Cryptococcus neoformans*, representatives of the order *Mucorales*),^2,23,24^ including multi-drug-resistant species (*Fusarium* species*, Scopulariopsis brevicaulis*, *Mucorales* species).^5,25,26^ To investigate antibacterial activity, typical Gram-positive and Gram-negative pathogens were used. Our results indicate a very selective antifungal activity of the new cationic β-glucans. We showed good activity against *S. brevicaulis*, which is important from a therapeutic point of view, as this fungus exhibits natural resistance to most of the antifungal drugs available today.^25,27−29^*S. brevicaulis* primarily causes onychomycosis in humans,^30^ but it has also been reported to cause deep infections, including disseminated mycoses.^26,31,32^ New options for treating infections caused by this fungus are therefore desirable. The obtained results constitute the basis for further research on the mechanism of action of new β-glucans on this species. We have also demonstrated the activity of polymers against *C. neoformans*, which is one of the most common human fungal pathogens responsible for meningitis and causes 1 million infections each year, mainly in HIV-infected patients.^33^ Fluconazole resistance has been reported for this species due to the frequent use of this drug in humans and as a plant protection agent.^34^

## Results

### Physicochemical Properties of the Obtained Polycations

The most important parameter from the point of view of the purity of the raw polysaccharide obtained (and the related biological activity) is the question of the presence of nitrogen atoms in the samples. Their high abundance indicates the presence of protein impurities in the material, which may have undesired biological properties. For both final polycation synthesis substrates, purification reduces the amount of nitrogen (Table S1) and, therefore, the amount of protein compared to the original sample. The fraction intended to contain these impurities is also significantly richer in nitrogen (Table S1). The final reaction, which involves adding a quaternary amine to the macromolecule, increases the amount of nitrogen, confirming successful modification. The relatively moderate increase may suggest a moderate degree of modification (Table S1). The IR spectra (Figures S1–S3) as well as the results from the elemental analysis confirm the attachment of quaternary amines to the structures obtained, which is indicated by the peak at 1485 cm^–1^. The overall shape of the spectra obtained is characteristic of polysaccharides, especially β-glucans.^35^ The spectra of cationic polymers obtained from β-glucans originating from two sources are substantially similar. The obtained ^1^H NMR spectra (Figures S5 and S6) confirm the conclusions from the IR spectra. The polymers have quaternary amines in their structure, and the structures of both macromolecules are significantly similar. This may suggest that within the framework of the used isolation method, the obtained compounds of this type will have a similar chemical structure regardless of the biological origin. The results described above are complemented by data obtained from GPC chromatography and ζ-potential measurements and the quantification of the degree of substitution with quaternary amines (Table 1) (details of the calculation is given in the Supporting Information Section 1.4), which characterize the compounds from the perspective of their macromolecular structure. These results confirm that two polycations (positive ζ-potential, Table 1) differing slightly in molecular weight were obtained with a very similar degree of modification.

**Table 1:** Physicochemical Characteristics of the Obtained Polymers

name of polymer	molecular weight *M*_n_ [kDa]	ζ-potential in water [mV]	degree of substitution with quaternary amines [% per glucose unit]
BBGGTMAC	84.57	38.17 ± 0.66	14.2 ± 0.8
OBGGTMAC	69.83	33.57 ± 0.42	14.8 ± 1.0

### Cell Toxicity

**Figure 1:**
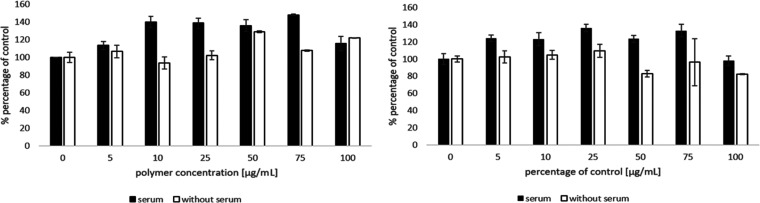
Effect of the resulting cationic β-glucan derivatives on the viability of 3T3-L1 cells in serum and serum-free medium. Left: BBGGTMAC; right: OBGGTMAC.

The obtained cationic β-glucan derivatives are intended to be used in the therapy of fungal diseases. From the point of view of such application, it is important to assess their toxicity toward mammalian cells. The cell line (3T3-L1) derived from mouse fibroblasts was used for preliminary toxicity assessment. This line is used extensively for this purpose^36^ and is the line representative of connective tissue, the most abundant tissue in the human body. The results obtained show that the BBGGTMAC polymer has no negative effect on the tested cell line in systems with and without serum (Figure 1). In the case of the second polymer OBGGTMAC, a similar effect is observed with the difference that in systems without serum for the highest concentrations, there is a slight decrease in the number of viable cells to a value not exceeding 80% (Figure 1). In the case of systems with serum, both polymers exhibit the proproliferative effect (Figure 1) previously observed for cationic polysaccharides.^37^ The general conclusion is that based on the results obtained, the new polymers are not toxic to mammalian cells.

### Impact of Polymers on Microorganisms

Table 2 presents the results of antimicrobial susceptibility testing showing the effect of the obtained polycations on 19 species of fungi and five species of bacteria (Gram-positive and Gram-negative). These species were chosen because they are representative of the most common human pathogens. In the case of bacteria, we observe a negligible effect, whether they are Gram-positive or Gram-negative. In some cases, only a reduction of the growth occurs for concentrations close to the maximum applied but not complete suppression of growth. The weak inhibitory effect of polymers was also observed in the case of yeasts. The best activity was recorded for *C. neoformans*, but it was still mainly the reduction of growth rather than a complete inhibition. A more significant antimicrobial effect was observed against filamentous fungi. We have demonstrated the antifungal activity of β-glucan derivatives against *S. brevicaulis*. The effect can be considered very significant, with MIC values for strain from commercial source *S. brevicaulis* DSM 9122 being 1.90 μg/mL for BBGGTMAC and 0.98 μg/mL for OBGGTMAC. Due to the good antifungal activity of tested β-glucans on *S. brevicaulis* in preliminary studies, three additional strains were investigated, and comparable results were obtained. The range of MIC values for BBGGTMAC and OBGGTMAC were 1.90–7.81 and 3.91–15.62 μg/mL, respectively. This demonstrates that the antimycotic activity of polycations against *S. brevicaulis* does not depend on the strain but is rather species-specific. Other filamentous fungi used in the studies were resistant to BBGGTMAC and OBGGTMAC and they grew well in the presence of these compounds. Only for *Aspergillus brasiliensis* and BBGGTMAC, we have observed growth impairment at a concentration of 15.61 μg/mL. It can be assumed that the better antifungal activity of BBGGTMAC than OBGGTMAC visible as lower MIC values for susceptible species is due to the higher toxicity of the first. The first polymer has a higher molecular weight, which is the main parameter differentiating those two tested compounds.

**Table 2:** Minimal Inhibitory Concentrations (MICs) of Tested β-Glucan Polymers against Fungal and Bacterial Species

	MIC [μg/mL]	
species	BBGGTMAC	OBGGTMAC
Filamentous Fungi		
*Scopulariopsis brevicaulis* DSM 9122	1.90	0.98
*Scopulariopsis brevicaulis* CM 65	1.90	3.91
*Scopulariopsis brevicaulis* CM 34	3.91	15.62
*Scopulariopsis brevicaulis* CM 39	7.81	7.81
*Aspergillus brasiliensis* ATCC 16404	>250 (15.61 impaired growth)	>250
*Aspergillus flavus* ATCC 204304	>250	>250
*Aspergillus fumigatus* DSM 819	>250	>250
*Aspergillus terreus* DSM 1958	>250	>250
*Fusarium oxysporum* DSM 841	>250	>250
*Fusarium solani* DSM 1164	>250	>250
*Mucor irregularis* CM P62	>250	>250
*Mucor pusillus* CM P32	>250	>250
*Mucor racemosus* CM P63	>250	>250
*Rhizopus oryzae* DSM 854	>250	>250
*Trichophyton interdigitale* DSM 4167	>250	>250
*Trichophyton mentagrophytes* ATCC 18748	>250	>250
*Trichophyton rubrum* DSM 16111	>250	>250
*Trichophyton tonsurans* DSM 12285	>250	>250
Yeasts		
*Cryptococcus neoformans* ATCC 204092	125 (7.8 impaired growth)	>250 (62.5 impaired growth)
*Candida albicans* ATCC 90028	>250	>250
*Candida glabrata* ATCC 15545	>250	>250
*Candida krusei* ATCC 6258	>250	>250
Bacteria Gram-Positive		
*Enterococcus faecalis* ATCC 29212	>250 (125 impaired growth)	>250
*Staphylococcus aureus* ATCC 29213 (Gram-positive)	>250 (125 impaired growth)	>250
Bacteria Gram-Negative		
*Escherichia coli* ATCC 25922	>250 (250 impaired growth)	>250
*Pseudomonas aeruginosa* ATCC 9027	>250	>250
*Salmonella enterica* ATCC BAA-664	>250	>250

### Studies on Model Membranes

#### Comparison of the Properties of Model Systems

**Figure 2:**
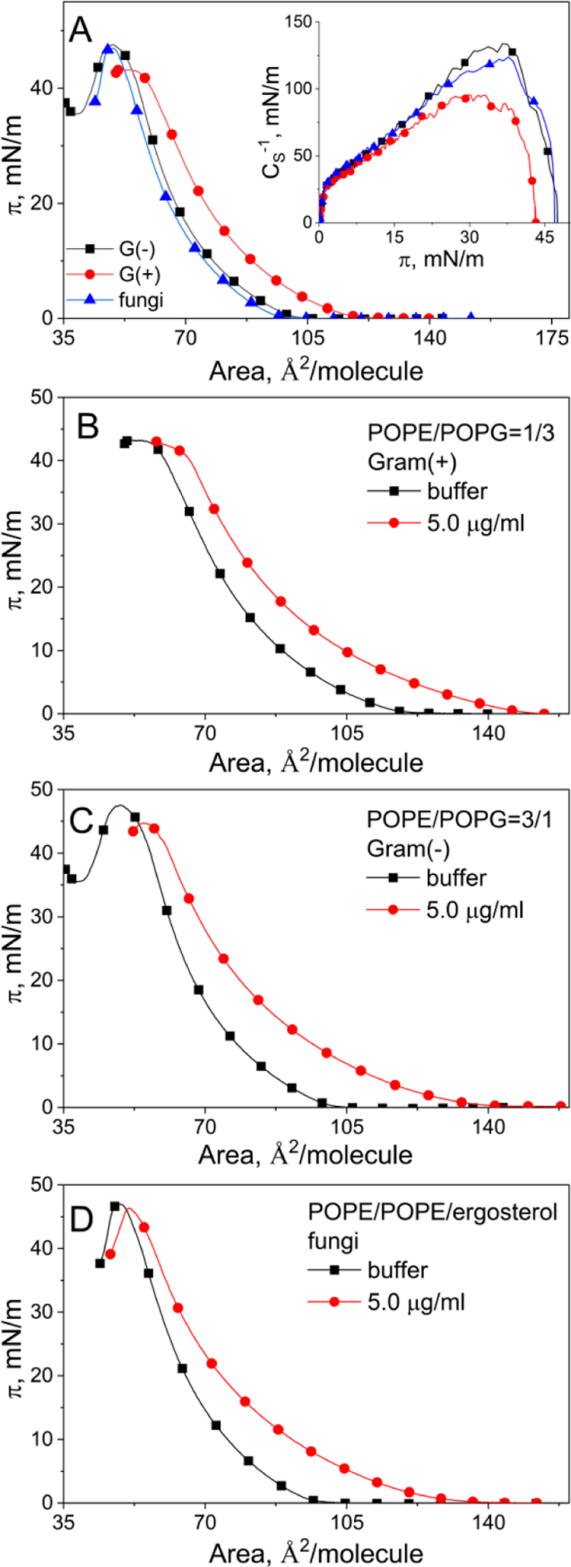
Isotherms for model membranes on buffer solutions; the isotherms for model membranes on BBGGTMAC polycation solution (A): Gram-positive bacterial (B), Gram-negative bacterial (C), and fungal (D) membranes.

In Figure 2A, the surface pressure–area curves (π–*A* isotherms) for the monolayers imitating bacterial and fungal membranes are shown. Based on these curves, the compressional modulus values were calculated according to eq 1(38)
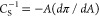
1where *A* is the mean area per molecule value at a given surface pressure π. The calculated values of the compressional modulus are presented in the inset of Figure 2A.

Based on the analysis of the isotherms and parameters obtained from these curves, the properties of monolayers can be compared. Namely, the steeper the curve and the lower the area per molecule at a given surface pressure, the more condensed the monolayer. The higher the collapse surface pressure, the larger the stability of the film. Additionally, the higher the values of *C*_S_^–1^, the larger the ordering in the monolayer. Based on the values of these parameters, the state of the monolayer can also be classified.^38^

As seen in Figure 2A, the isotherms recorded for Gram-negative bacterial and fungal membranes are of very comparable shape and position. A similar value of the area per molecule at a given surface pressure and nearly the same collapse surface pressure value indicates that both these models exhibit comparable condensation and stability. For both of them, the values of *C*_S_^–1^ are also similar, indicating comparable ordering of molecules in these films. The maximal value of this parameter allows one to classify their state as liquid condensed (LC). To summarize, although the monolayers imitating Gram-negative and fungal membranes are of different lipid compositions, they are of similar condensation, ordering, and stability.

The curve for the Gram-positive bacterial membrane model is localized at larger areas, it collapses at lower surface pressure, and the *C*_S_^–1^ values for this film are lower than those obtained for the remaining membranes. All of these indicate that this monolayer is less condensed, less ordered, and less stable than the remaining model systems.

#### Influence of the BBGGTMAC Polymer on Model Membranes

The studies were performed only for the BBGGTMAC polymer, which exhibited better *in vitro* antimicrobial activity. The monolayers imitating particular membranes were spread on polymer solutions with concentrations of 1, 2.5, and 5.0 μg/mL. For the clarity of presentation, only the curves recorded on the highest concentration of polymer are shown in Figure 2B–D. As can be noticed for all of the systems, the curves recorded on polymer solution are shifted toward larger areas, and they are less steep than the isotherms recorded on the buffer. The shift was observed even at the lowest concentration of polymer applied in the experiment (1.0 μg/mL); however, it was rather weak. The shift means that BBGGTMAC polymer molecules decrease the condensation and ordering in the monolayers. Moreover, the films formed on the subphase containing a higher concentration of polymer collapse at lower surface pressure; thus, in the presence of the polymer, the monolayer is less stable. This effect is not observed for the system imitating Gram-positive bacterial membrane.

**Figure 3:**
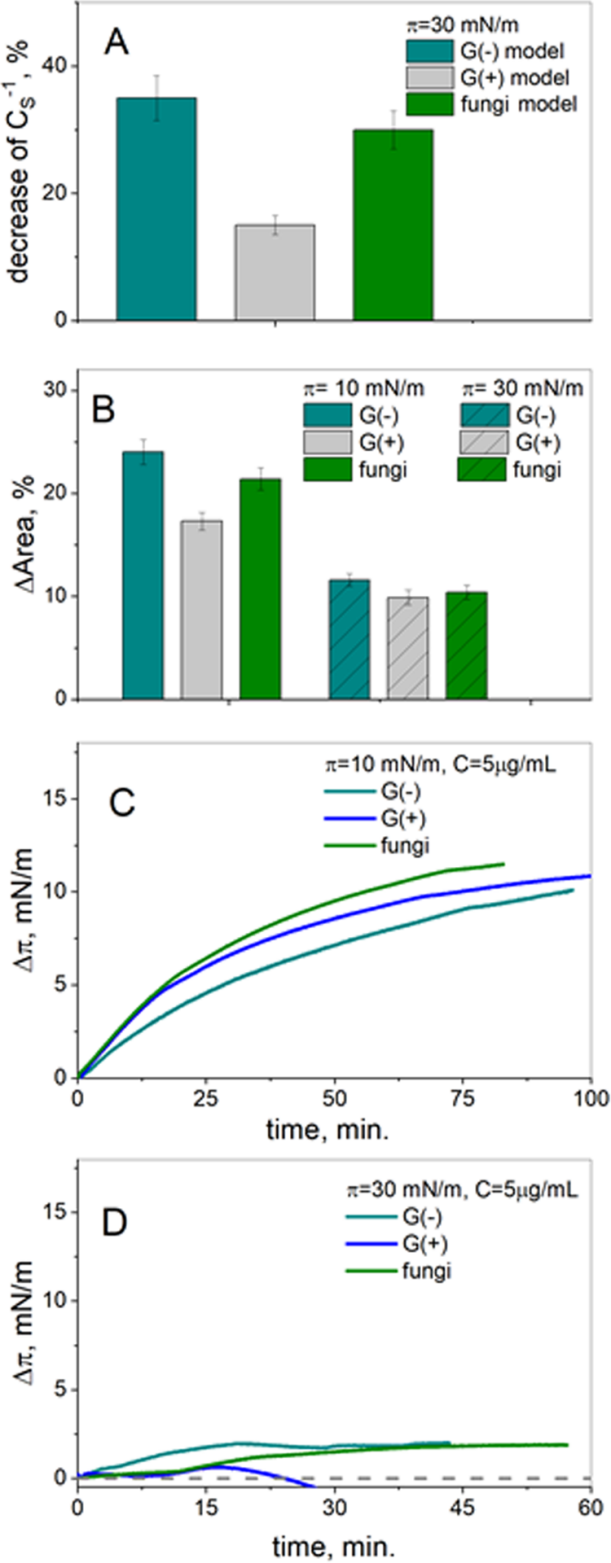
Drop of compressional modulus (A) and the shift of the isotherms for model membranes on BBGGTMAC polycation solution (B); the penetration of polycation molecules into model membranes at low (C) and high (D) surface pressure.

To compare the effect of BBGGTMAC on monolayers more precisely, further calculations on particular films were done. Namely, the compressional modulus values were calculated according to eq 1. Then, the percentage decrease of the values of this parameter caused by the polymer at its given concentration was calculated and presented for one surface pressure, π = 30 mN/m (Figure 3A). Similarly, the shift of the isotherms with respect to the curve on the buffer at low (10 mN/m) and high (30 mN/m) surface pressure was calculated and is presented in Figure 3B.

From the analysis of Figure 3 results, it could be concluded that the studied polymer exerts the weakest effect on the Gram-positive bacterial membrane model. For Gram-negative bacteria and fungal membranes, both the decrease of the compressional modulus values and the shift of the curves are comparable in the range of error. The latter corresponds with strong similarities in the condensation, ordering, and stability of Gram-negative bacterial and fungal membranes postulated above. Moreover, it is evident that the shift of the isotherm is larger at lower surface pressures, that is, for less-compressed monolayers.

On the other hand, the Gram-positive bacterial membrane model exhibits much lower condensation and ordering than the remaining membrane systems, and simultaneously it is significantly less sensitive to the influence of polymer. This allows one to assume that not (only) the parameters of the model system but rather its composition is important from the point of view of the effect of polymer.

The penetration results presented as the changes in the surface pressure value after injection of polymer (Δπ) with time are shown in Figure 3C,D. In short, the increase of Δπ with time means that the molecules penetrate the monolayer. A decrease of this parameter indicates desorption of the injected molecules from the interface, while Δπ = 0 means no penetration or full desorption. Sometimes the surface pressure decreases below the value of the injection surface pressure, and thus Δπ values are negative. This situation suggests dragging of a monolayer material to the subphase, leading to destabilization of the film.^13^

As observed in Figure 3C,D, the Δπ values are significantly higher at lower surface pressure. The latter results from lower condensation of model systems at these conditions, facilitating the incorporation of molecules into the film. Furthermore, at π = 10 mN/m, BBGGTMAC polymer molecules are incorporated into all of the studied model membranes, which is accompanied by an increase of the surface pressure with time. As seen in Figure 3C, π values increase systematically with time, and they are slightly higher for fungal membranes as compared to bacterial membranes.

The results obtained at π = 30 mN/m are more complicated. Namely, the injection of polymer into the Gram-positive bacterial membrane model system initially causes some negligible increase of the surface pressure. However, in a short time, the molecules are desorbed from the interface together with the monolayer materials. For Gram-negative bacterial and fungal membranes, the increase of the surface pressure is noticed. Thus, the polymer penetrates both model systems. Initially, the penetration is stronger for bacterial membranes (this is manifested in the increase of π within the first few minutes of experiments); however, with time, the incorporation becomes very similar in both types of membranes.

#### Influence of the BBGGTMAC Polymer on One-Component Lipid Monolayers

**Figure 4:**
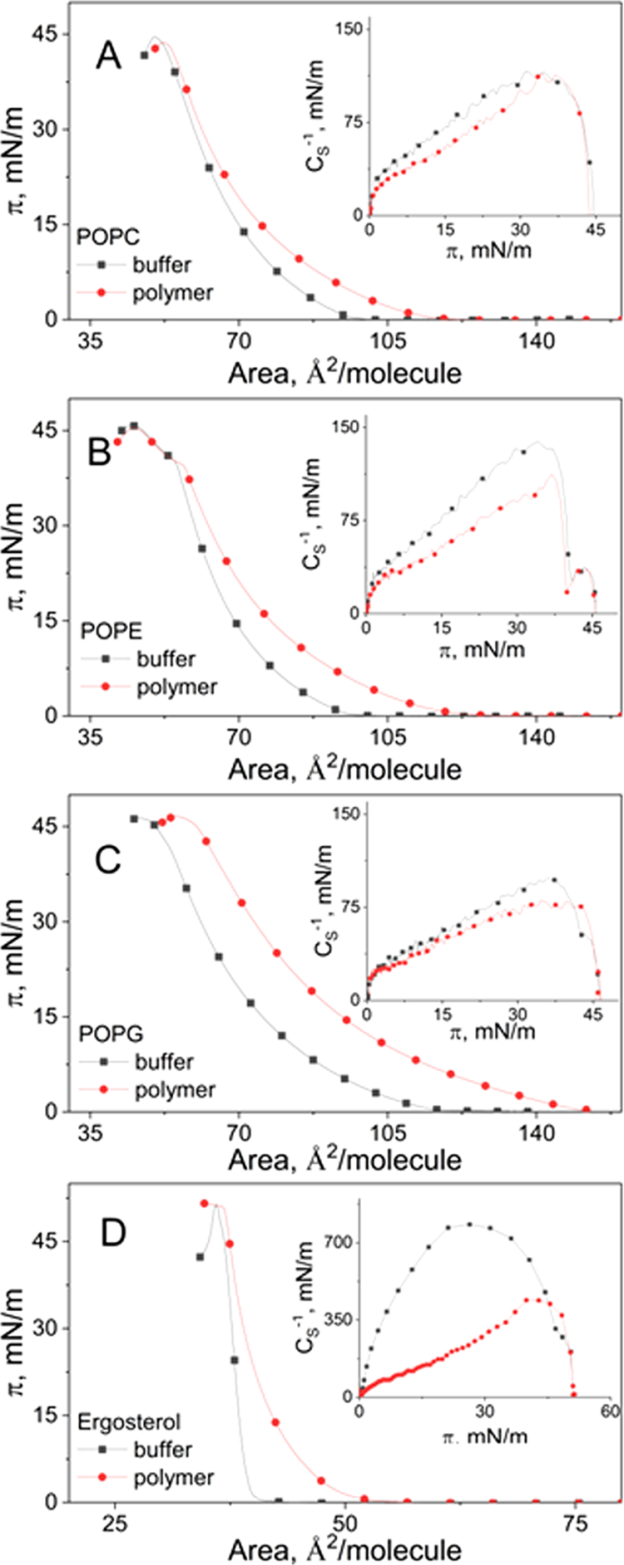
Isotherms for phospholipids and ergosterol on buffer and on BBGGTMAC polycation solution; insets: the compressional modulus vs the surface pressure plots for particular monolayers.

To analyze the possible correlation between the composition of the model system and the effect of polymer, the investigations for one-component monolayers formed by the lipids used for the formation of model membranes were done. The isotherms on buffer and polymer solutions are shown in Figure 4.

Comparison of the isotherms on buffer revealed that phospholipid molecules, namely, POPC, POPE, and POPG, form more expanded monolayers than sterol. Among the studied phospholipids, the films of POPG are less condensed than the remaining films. This relates to the structure of the PG polar head carrying a negative charge and preventing a tight packing of the molecules. Phosphatidylcholines and phosphatidylethanol amines at the applied experimental conditions are zwitterions, and in a wide range of surface pressure, they form films of comparable condensation. However, for POPE with π > 35 mN/m, a phase transition appears. And finally, ergosterol is known to form highly condensed and ordered films, which reflects in the steepness of the isotherm and high compressional modulus values.

In the presence of BBGGTMAC polymer molecules, the curves are shifted to larger areas, and they have a more expanded shape as compared to the film on buffer. Also, a decrease of the compressional modulus values can be detected.

Thus, BBGGTMAC makes the lipid monolayer less closely packed and less ordered. Among phospholipids, this effect decreases in the following order: POPG > POPE > POPC. However, interesting results were obtained for the ergosterol film. Namely, polymer significantly decreases its condensation and ordering despite the extremely high packing and ordering of this monolayer. This additionally confirms the thesis that not only the organization of the model system but also its composition determines the effect of the polymer.

**Figure 5:**
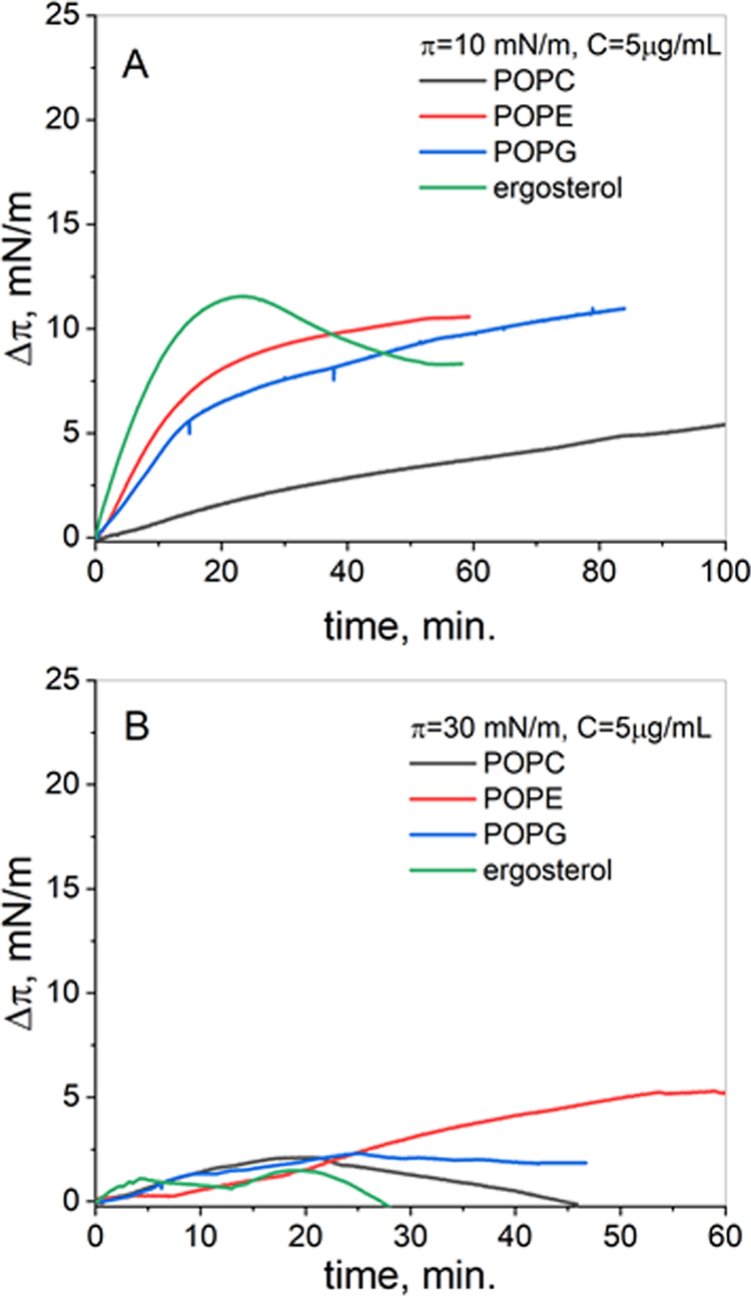
Penetration of BBGGTMAC polycation molecules into lipid monolayers at low (A) and high (B) surface pressure.

Also, the penetration results (Figure 5) evidence that polymer molecules show a strong affinity to ergosterol films. At low surface pressure, they incorporate into sterol films the most significantly among the studied lipids in a short time.

At high surface pressure during the first 20 min of measurements, the Δπ values achieved after the injection of polymer are comparable for all of the monolayers. Then, for ergosterol and POPC, they decrease below zero. For POPE, the increase of penetration with time can be observed. These results evidence that ergosterol is important from the point of view of interactions with the studied polymer. In model fungal membrane, this lipid is in the mixture with POPC and POPE, which together form the environment-facilitated incorporation of polymer molecules. This is reflected in the data collected for mixed systems. The second lipid, considering its interactions with polymer, namely, POPE, is an important component of all of the studied systems. In Gram-negative bacterial membranes, it prevails strongly on POPG, which may explain the stronger effect of the polymer on this group of bacteria.

## Discussion

The first subject of this work was the extraction of two β-glucans from barley and oats and the modification of their structure by the addition of a quaternary amine (by a reaction with aminoepoxide GTMAC) to obtain a stable polycation. The effectiveness of these processes was confirmed by spectroscopic methods (FTIR and ^1^H NMR spectra) and elemental analysis (combustion analysis). Characterizations of macromolecules were completed by determination of their molecular weights (GPC/SEC chromatography), degrees of modification (precipitation titration of chlorides being counterions here), and ζ-potentials of particles in solution. The obtained compounds were used to assess the toxicity of novel structures of this type toward cells of various organisms and to explain the mechanisms of interaction responsible for this phenomenon.

The answer to the question about the mechanism responsible for the toxicity of the polycations toward microorganisms and mammalian cells is, as expected, complex. According to our results, this is not a universal effect that is beneficial and suggests that such systems could be used to develop selectively active antimycotics medication. There is practically no negative effect on fibroblasts in the range of tested concentrations (up to 100 μg/mL), which is a desirable effect, and suggests good values of therapeutic indexes (their precise determination would require further studies beyond the scope of this work). However, high toxicity was observed for selected pathogenic microorganisms. The strongest negative effects were observed for filamentous fungi *S. brevicaulis* and yeasts *C. neoformans*, while the antimicrobial effect was insignificant for other tested fungi and five bacterial species tested (Gram-positive and Gram-negative).

Another aspect investigated was the mechanism responsible for this selective toxicity of the new compounds obtained. Antimicrobial drugs have different mechanisms of action and can target cell wall, protein biosynthesis, inhibition of DNA replication,^39^ as well as interaction with membranes.^38^ For positively charged polymer substances like ours tested, interaction with negatively charged membranes appears to be one of the likely mechanisms of action. This mode of action is known with some antifungal drugs such as polyenes, i.e., amphotericin B and nystatin, which exactly target ergosterol in the plasma and vacuole membrane.^40,41^ Therefore, this aspect of new systems was investigated in this work as a potential mechanism of action. To carry out these studies, *ex vivo* models of biological membranes representative of the most important families of microorganisms (fungi, Gram-positive and Gram-negative bacteria) were prepared, and the effects of the most promising polycation obtained on them were studied using Langmuir monolayers. The most important finding from these experiments is that the effect of polymer is not determined solely by the system molecular organization (condensation, ordering, stability), but the model composition is of high importance. Among the studied lipids, ergosterol seems to be the molecule of special interest in further experiments. The results of model membrane experiments evidenced a high affinity of the studied polymer to the monolayers containing ergosterol (model fungal membrane) and for one-component ergosterol film. These results correlate well with biological studies, in which a toxic effect of polymer on selected filamentous fungi and yeast was confirmed. However, probably not the presence of ergosterol itself but also its concentration in the membrane determine the toxic effect of the studied polycation. The latter may explain why some fungal species were found to be insensitive to the action of polycation. The issues of the correlation between the level of ergosterol in the system and the toxicity of polycation should be investigated in the future. And finally, the studied polymer is a polycation, which allows one to predict its stronger affinity to the systems composed of the negatively charged lipids (bacterial membrane). Although the polymer exerts a fluidizing effect on POPG films and POPG-containing model bacterial membranes, the monolayer experiments do not indicate the latter relationship. Namely, the model system dominated by the negatively charged POPG was less susceptible to the effect of polycation than the model bacterial membrane system of lower POPG content. Moreover, *in vitro* studies also evidenced that the bacterial membranes composed of negatively charged PG molecules are less susceptible to the investigated polymer than fungi. The latter additionally confirmed the importance of ergosterol in the mechanism of action of the studied polymer. On the other hand, the monolayer experiments allow one to predict the toxicity of polymer to Gram-negative bacteria. Unfortunately, the latter does not correlate with the results of biological experiments, which evidenced that the antimicrobial effect of polymer on bacteria was rather insignificant.

The results obtained on model systems are in acceptable agreement with those obtained for biological experiments and indicate that the nature of the toxic effect can be related to the biological membrane. The lack of a full correlation of the antifungal studies and studies on model membranes may be related to effects arising from the presence of the cell wall, which screens the membrane of bacterial and fungal cells and might limit the interaction of the polycations. The barrier between the environment and the interior of the cell seems to be crucial here, and its variations across species are presumably the basis for the selectivity of the observed processes. The diversity of the fungal cell wall is a known phenomenon. The differences in the composition of the cell wall in fungi are visible not only at the genus and species level^42^ but also between individual strains of the same species. Moreover, differences were also described for one individual strain. The studies by Bleichrodt *et al.*(43) showed that *A. fumigatus* cell wall composition is heterogeneous between single conidia and also changes during germination. Changes in the cell wall composition were reflected in susceptibility to caspofungin.^43^

## Experimental Data

### Isolation of β-Glucans and Synthesis of New Polymers

Wholemeal barley flour and wholemeal oat flour were used, both of which originated at Gospodarstwo Rolne Mateusz Gren Radcze Farm in Poland. Ethanol (96%, p.a.), HCl (36%, p.a.), NaCl (p.a.), Na_2_SO_4_ (p.a.), and acetic acid (99%) were bought from Chempur (Piekary Slaskie, Poland). Glycidyltrimethylammonium chloride (GTMAC, 50% in water) was bought from Sigma-Aldrich (Burlington, MA). Dialysis membrane Spectra/Por MWCO 3500 Cal Roth (Karlsruhe, Germany) was used. In cases of all polymers for GPC/SEC measurements, the column PolySep-SEC GFC-P Linear, LC Column 300 × 7.8 mm^2^ (Phenomenex, Torrance, CA) was used.

### Model Membranes (Langmuir Monolayers)

In Langmuir monolayer experiments, the synthetic lipids of high purity (≥99%) purchased from Avanti Polar Lipids Inc. were used: 1-palmitoyl-2-oleoyl-*sn*-glycero-3-phosphoethanolamine (POPE), 1-palmitoyl-2-oleoyl-*sn*-glycero-3-phospho-(1′-rac-glycerol) (sodium salt) (POPG), 1-palmitoyl-2-oleoyl-*sn*-glycero-3-phosphocholine (POPC), and ergosterol. The stock solutions of POPE, POPG, and POPC were prepared in the mixture of chloroform/methanol (9:1 v/v) (both solvents were of HPLC grade, ≥99.9%, Aldrich). Ergosterol was dissolved in pure chloroform. The salts used for the preparation of phosphate-buffered saline (PBS, pH = 7.4), namely, sodium chloride, potassium chloride, disodium hydrogen phosphate, and potassium dihydrogen phosphate (a purity >99%), were supplied by POCH S.A. The polymer chosen for these experiments was obtained according to the procedure described in the Cell Toxicity section, and its solutions were prepared in PBS buffer. In the experiments, ultrapure Milli-Q water was used.

### Biological Experiments

For cell culture, 3T3-L1 (ATCC CL-173) cells purchased from American Type Culture Collection (ATCC) (Manassas, VA) were used. Cell medium (DMEM) and additives were purchased from Sigma-Aldrich (Burlington, MA).

For antimicrobial activity testing, strains from open culture collection ATCC and Deutsche Sammlung von Mikroorganismen und Zellkulturen (DSMZ) were used, as well as strains from Chair of Microbiology, Jagiellonian University Medical College, Krakow, Poland. The following species were used in the study: bacteria: *Escherichia coli*, *Enterococcus faecalis*, *Salmonella enterica*, *Staphylococcus aureus*, and *Pseudomonas aeruginosa*; yeasts: *Candida albicans*, *Candida glabrata*, *Candida krusei*, and *Cryptococcus neoformans*; filamentous fungi: *Aspergillus brasiliensis*, *Aspergillus flavus*, *Aspergillus fumigatus*, *Aspergillus terreus*, *Fusarium oxysporum*, *Fusarium solani*, *Mucor irregularis*, *Mucor pusillus*, *Mucor racemosus*, *Rhizopus oryzae*, *Scopulariopsis brevicaulis*, *Trichophyton interdigitale*, *Trichophyton mentagrophytes*, *Trichophyton rubrum*, and *Trichophyton tonsurans*.

Fungal strains were cultured on Sabouraud potato dextrose agar (Graso Biotech, Poland), potato dextrose agar (Graso Biotech, Poland) and Czapek yeast agar (ingredients per 1 L of medium: 1 mL of Czapek concentrate, 1 g of K_2_HPO_4_ (POCH, Poland), 5 g of yeast extract (Merck), 30 g of saccharose (Chempur, Poland), and 16 g of agar (Graso Biotech, Polska)). The Czapek concentrate was prepared from the following ingredients from POCH, Poland, per 50 mL of concentrate: 15 g of NaNO_3_, 17.5 g of KCl, 2.5 g of MgSO_4_·7H_2_O, 0.05 g of ZnSO_4_·7H_2_O, and 0.025 g of CuSO_4_·7 H_2_O. Bacteria were cultured on tryptic soy agar (BD Difco).

Bacterial and yeast inocula were prepared in sterile distilled water; for filamentous fungal inocula, sterile distilled water with Tween 20 (Sigma-Aldrich) was used.

For antimicrobial activity testing, the following materials were used: liquid RPMI-1640 medium with l-glutamine, without sodium bicarbonate (Sigma-Aldrich), supplemented with 2% glucose (Chempur, Piekary Slaskie, Poland), buffered to pH 7 with 4-morpholinepropanesulfonic acid (MOPS) (Glentham Life, Corsham, U.K.) (0.165 mol/L) for fungi, and Mueller Hinton Broth (Sigma-Aldrich) for bacteria.

### Isolation of β-Glucans

Isolation of β-glucans from barley and oat flour was carried out according to the procedure described previously in the literature.^44^ Briefly, 20 g of barley or oat flour was suspended in 200 mL of water. The pH of the obtained mixture was adjusted to 7.6 with a 10% aqueous solution of potassium carbonate and heated for 30 min at 45.5 °C. The suspension was then centrifuged for 30 min at 4 °C and 4940*g* to obtain the supernatant. The resulting clear solution was acidified to pH 4.5 with 2M HCl and centrifuged again for 30 min at 4 °C and 4940*g* to collect the supernatant. The collected solution was precipitated with ethanol (volume ratio 1:1 supernatant/ ethanol) and left for 12 h at 8 °C to settle out. The mixture was then centrifuged for 10 min at 3780*g*, and the resulting precipitate was dried under reduced pressure at 40 °C. Barley β-glucan (0.62 g, extraction yields 3.1%) and 0.6 g of oat β-glucan (extraction yields 3.1%) were obtained. The isolation yields calculated are subject to large uncertainty due to the unknown amount of water in the flour and are not fully credible.

### Cationization of β-Glucans

β-glucan was obtained at the isolation step; 0.62 g of barley or 0.6 g of oat was suspended in 50 mL of water with 200 mg of NaOH at 60 °C while stirring the mixture for 30 min. Then, 6 mL of GTMAC was added with continuous stirring. The reaction was carried out at 60 °C for 4 h, and after this time, the solution was transferred to a dialysis tube and dialyzed against water for 4 days while changing the water to fresh one once a day. The obtained clear mixture was centrifuged for 5 min at 10 000*g*, and the supernatant was freeze-dried to isolate the polymer from the solution. Barley polycation (0.53 g, 85% efficiency) and 0.48 g of oat polycation (80% efficiency) were obtained. ^1^H NMR (5 g/L solution in D_2_O) and FTIR (ATR) spectra were performed for all of the obtained polymers.

### Determination of the Degree of Substitution of Polysaccharide by Quaternary Amines

The degree of hydroxyl group substitution in the polysaccharide by GTMAC addition was determined using conductometric precipitation titration.^40^ The measurement consisted of determining the amount of chloride ions proportional to the number of amines attached to the macromolecule per unit of glucose. For this purpose, a 0.017 M solution of AgNO_3_ and a 1 g/L solution of the corresponding polycation were prepared. AgNO_3_ (0.25 mL) was added to 10 mL of polymer solution, and conductivity was measured after each step. Initially, the conductivity decreased with the addition of AgNO_3_ solution (see Figure S7 for exact data). This is due to the precipitation of AgCl, whereas after the utilization of all chloride ions, the conductivity started to increase. The volume corresponding to the change in trend determined from the point of intersection of the two tangents to the course of change allows the degree of modification to be calculated from the corresponding formula (Supporting Information Section 1.4).^45^

### Determination of the Molecular Weight of Polymers by Gel Permeation/Size-Exclusion Chromatography

The eluent consists of 0.5 M acetic acid and 0.3 M Na_2_SO_4_ in aqueous solution, the flow rate was 0.8 mL/min, and the injection rate was 100 μL for all samples.^46^ The polymers were dissolved in the eluent at a concentration of 5 g/L. Because of the positive charge of these polymers (confirmed by measurements of ζ-potential), poly(2-vinylpyridine) standards were used for molecular mass determination.

### ζ-Potential Measurements of the Obtained Polycations

The measurement of the ζ-potential was performed using a Zetasizer Nano ZS, and the polymer concentration was 5 g/L; demineralized water (pH 6.0, conductivity 0.5 μS) was used as a solvent. Three independent measurements were made for each sample; the result presented is the arithmetic mean ± standard deviation (SD) of the obtained results.

### Toxicity Testing of Polymers on Cell Lines

Embryo mouse fibroblast 3T3-L1 was used to assess the toxicity of the new polymers obtained. Dulbecco’s modified Eagle’s medium, supplemented with fetal bovine serum, for a final concentration of 10% (v/v) and 1% (v/v) penicillin–streptomycin solution was used. Cultures were incubated at 37 °C in an atmosphere containing 5% carbon dioxide. For each experiment, the cells were seeded at 6 × 10^4^ per well into 24-well plates and grown for 24 h. After that, the medium was changed to full medium (containing 10% fetal bovine serum) or serum-free, and polycation solution (in serum-free media) was added for the next 24 h to assess cytotoxicity using the crystal violet assay.^47^ Three independent measurements were made for each concentration, and the result presented is the arithmetic mean ± standard deviation (SD) of the obtained results.

### Antimicrobial Properties of Polymers

We have investigated the antifungal and antibacterial activity of modified β-glucans in *in vitro* experiments. The method used was based on European Committee on Antimicrobial Susceptibility Testing (EUCAST) procedures for antifungal susceptibility testing of fungi to antimycotic drugs^48,49^ and the Hancock Lab procedure for cationic antimicrobial peptides.^50^ Generally, microorganisms were cultured in a liquid media (RPMI-1640 medium with l-glutamine, without sodium bicarbonate, supplemented with 2% glucose and buffered to pH 7 with 4-morpholinepropanesulfonic acid (MOPS) (0.165 mol/L) (later referred to as RPMI) for fungi and Mueller Hinton Broth II for bacteria (later referred to as MHB)) in flat-bottom polypropylene 96-well microdilution plates (VWR, Radnor, PA) in the presence of various concentrations of tested polymers.

β-glucans were dissolved in sterile distilled water to obtain a concentration of 5 g/L. From stock solutions, a series of twofold dilutions in water were made, resulting in a concentration range of 4.9–2500 μg/mL. Wells 1–10 of each column of microdilution plates were filled with 20 μL of the corresponding concentration of tested polymers. Wells of columns 11 and 12 were the control of microbial growth and the control of sterility, respectively, and were filled with 20 μL of sterile distilled water instead of polymers.

Fungi were cultured on Sabouraud glucose agar with chloramphenicol, Czapek yeast extract agar, and potato dextrose agar to obtain optimal growth and sporulation. Bacteria were cultured on tryptic soy agar for 24 h. Yeast and bacterial inocula were prepared by suspending a few representative colonies in sterile distilled water. Filamentous fungi colonies were covered with ∼5 mL of sterile water supplemented with Tween 20, then the conidia were rubbed with a sterile cotton swab, and the suspension was transferred to a sterile tube attached to a sterile filter with a pore diameter of 10 μm. The suspension was filtered to remove hyphae and collected. In some cases, double filtration with 10 and 20 μm filters was performed. Microbial suspensions were mixed with a vortex mixer (Labnet, Poland), and the cell density was adjusted to 0.5 McFarland with an automatic densitometer (Biosan, Poland). The obtained suspensions were diluted in liquid culture media: fungi 1:20 in RPMI and bacteria 1:200 in MHB. This gave the following inocula densities: 0.5–2.5 × 10^5^ CFU/mL for yeasts, 1–2.5 × 10^5^ CFU/mL for filamentous fungi, and 0.5–1 × 10^6^ CFU/mL for bacteria.

Wells 1–11 of each column of microdilution plates were inoculated with 180 μL of the microbial suspensions in culture media. Well 12 contained 180 μL of microbial-free medium (RPMI for fungi and MHB for bacteria). The addition of microorganisms resulted in a 10-fold dilution of the tested polymers. Thus, the final concentration range of β-glucans on microdilution plates was 0.49–250 mg/L.

The plates were incubated without agitation at 37 or 27 °C (incubator POL-EKO, Poland), depending on the species, in ambient air for 24–72 h. The antimicrobial activity was estimated visually by determining the minimal inhibitory concentration (MIC) values, which were defined as no visible growth of microorganisms by the eye and for yeasts and bacteria also with a microplate reader (Sunrise, Tecan Group Ltd., Männedorf, Switzerland) at an optical density of 530 nm.

### Interaction of a Polycation with a Model Biological Membrane

The Langmuir monolayer technique was applied to prepare and investigate three model systems of membranes, that is, Gram-positive bacterial membrane, Gram-negative bacterial membrane, and fungal membrane. The composition of these model systems was estimated based on the data on the lipid composition of bacterial and fungal membranes presented in the literature. In short, bacterial membranes are dominated by three classes of phospholipids, namely, zwitterionic phosphatidylethanol amine (PE), negatively charged phosphatidylglycerol (PG), and cardiolipin. Generally, Gram-negative bacteria as compared to Gram-positive species contain more PE in membranes. On the other hand, in Gram-positive bacteria, the negatively charged lipids prevail substantially over zwitterionic lipids.^51−54^ The model systems imitating bacterial membranes used in our studies were as follows: POPE/POPG = 3:1 (the molar ratio) mixture (model for Gram-negative bacteria) and POPE/POPG = 1:3 (the molar ratio) (model for Gram-positive bacteria).

The composition of the fungal membrane is more complicated as compared to the composition of the bacterial membrane, and it is also more strongly dependent on the fungus and determined by the stage of development and environmental conditions.^54−57^ However, the major phospholipids forming fungal membranes are phosphatidylcholines (PCs) and phosphatidylethanol amines (PEs).^57^ Moreover, in fungal membranes, the sterol (ergosterol) is also a very important lipid.^54−56^ Taking the foregoing into consideration in this work, the model of fungi contained POPC and POPE (1:1) and 10% of ergosterol.

### Monolayer Experiments

#### Surface Pressure (π)–Area (*A*) Measurements

The monolayers formed by particular lipids (one-component films) and their appropriate mixtures (model membranes) were spread on PBS (pH = 7.4) and on polymer solutions (prepared in PBS buffer), and the surface pressure (π)–area (*A*) isotherms during the monolayer compression were recorded.

The monolayers were formed by deposition of the lipid solutions on the subphase with the Hamilton microsyringe (± 1.0 μL). Then, the monolayers were left for 10 min before the compression was started with a barrier speed of 10 cm^2^/min. These experiments were done on the KSV-NIMA Langmuir–Blodgett trough (total area = 275 cm^2^) having two Delrin barriers, enabling symmetrical compression of the films. The trough was placed on an antivibration table. The surface pressure was measured (±0.1 mN/m) with the Wilhelmy plate made of filter paper (ashless Whatman Chr1) connected to an electrobalance. The error for the area per molecule does not exceed 0.2 Å^2^/molecule. The experiments were done at 20 °C, and the subphase temperature was controlled thermostatically (±0.1 °C) by a circulating water system.

#### Penetration Experiments

The penetration experiments were performed using the same Langmuir–Blodgett trough as it was applied in the surface pressure (π)–area (*A*) measurements. The experiments were done according to the following procedure. The monolayers of model systems and particular lipids were spread on the buffer subphase, and then they were compressed up to the target surface pressure and left for equilibration to desirable initial surface pressure π_i_ (π_i_ = 10 and 30 mN/m). Then, the solution of polymer was injected into the subphase, and the changes in the surface pressure (at a constant area) were monitored (π_exper._). The concentration of polymer after injection into the subphase was 5 μg/mL. During experiments, the subphase was continuously stirred. The results of these experiments were analyzed with respect to the initial surface pressure (π_i_), namely, the Δπ values (Δπ = π_exper_ – π_i_), were calculated and plotted as a function of time.

## Supporting Information Available

FTIR, ^1^H NMR spectra, and elemental analysis of the purification steps and final cationic β-glucans, and data concerning the determination of the degree of cationic modification of the polysaccharide (PDF)
