# Supplementary material for: Preliminary Studies on the Mechanism of Antifungal Activity of New Cationic β-Glucan Derivatives Obtained from Oats and Barley
Source: ACS Omega. 2022 Oct 27;7(44):40333–43. doi: 10.1021/acsomega.2c05311 (PMC9648169; doi:10.1021/acsomega.2c05311)
Supplement: Supplementary file 1 — ao2c05311_si_001.pdf [file ao2c05311_si_001.pdf]

Supporting Information

Preliminary studies on the mechanism of antifungal activity of new cationic beta-glucan derivatives obtained from oats and barley

Kamil Kamiński[1], Katarzyna Hąc-Wydro[1], Magdalena Skóra[2], Małgorzata Tymecka[1], Magdalena Obłoza[1]

1. Faculty of Chemistry, Jagiellonian University, Gronostajowa 2 St., 30-387 Kraków, Poland

2. Department of Infections Control and Mycology, Chair of Microbiology, Jagiellonian University Medical College, Czysta 18 St., 31-121 Kraków, Poland

1. General physical and chemical characteristics of polymers

1.1. Elemental composition of substrates and products of purification and modification based on combustion analysis.

Isolation of beta-glucans from cereal material involved selective extraction of this polymer and separation of protein impurities from the soluble fraction. The effectiveness of this process and the subsequent cationisation of the polysaccharides can be effectively monitored by examining the elemental composition of the materials obtained. Table S1 shows the results obtained for the most important isolation and synthesis steps.

Table S1. Elemental composition (nitrogen, carbon and hydrogen) and the ratio of nitrogen to carbon for substrates and products of isolation and synthesis.

	**Elemental composition [%]**			
	**N**	**C**	**H**	**N/C**
**Barley flour (BF)**	2.22 ± 0.13	41.83 ± 0.22	6.32 ± 0.01	0.053
**Protein barley residue (PBR)**	10.48 ± 0.36	47.16 ± 0.06	6.84 ± 0.01	0.222
**Barley beta-glucan (BBG)**	1.77 ± 0.07	40.82 ± 0.58	6.19 ± 0.03	0.043
**Barley beta-glucan modified by GTMAC (BBGGTMAC)**	3.71 ± 0.01	42.35 ± 0.03	7.03 ± 0.04	0.088
**Oat flour (OF)**	1.86 ± 0.06	42.40 ± 0.13	6.39 ± 0.07	0.044
**Protein oat residue (OBR)**	13.24 ± 0.07	47.25 ± 0.03	6.27 ± 0.08	0.280
**Oat beta-glucan (OBG)**	0.30 ± 0.01	35.10 ± 0.12	6.21 ± 0.09	0.008
**Oat beta-glucan modified by GTMAC (OBGGTMAC)**	2.31 ± 0.13	41.49 ± 0.23	6.55 ± 0.16	0.056

The most important parameter from the point of view of the purity of the polysaccharide obtained (and the related biological activity) is the question of the presence of nitrogen atoms in the samples. Their high abundance indicates the presence of proteins in the material which may have undesired biological

properties. For both of these materials, purification reduces the amount of nitrogen and therefore the amount of protein compared to the original sample, the fraction intended to contain these impurities is significantly richer in nitrogen. The cationisation process, which involves adding a quaternary amine to the macromolecule, on the contrary increases the amount of nitrogen, confirming successful modification. The relatively moderate increase may suggest a moderate degree of modification.

1.2. Infrared spectra of the compounds obtained (FTIR)

The spectra below (Figure S1) present the final cationisation product for barley and oat beta-glucans. IR spectra were also performed for raw flours of protein impurities and unmodified beta-glucans. These spectra can be found in Figures S2 and S3.

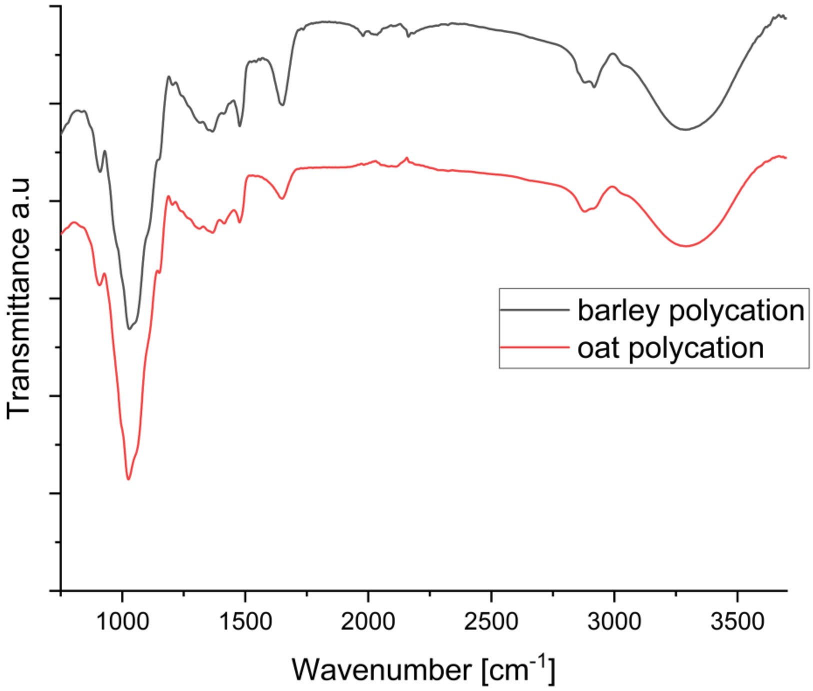

Figure S1. FTIR spectra of the new cationic beta-glucan derivatives obtained.

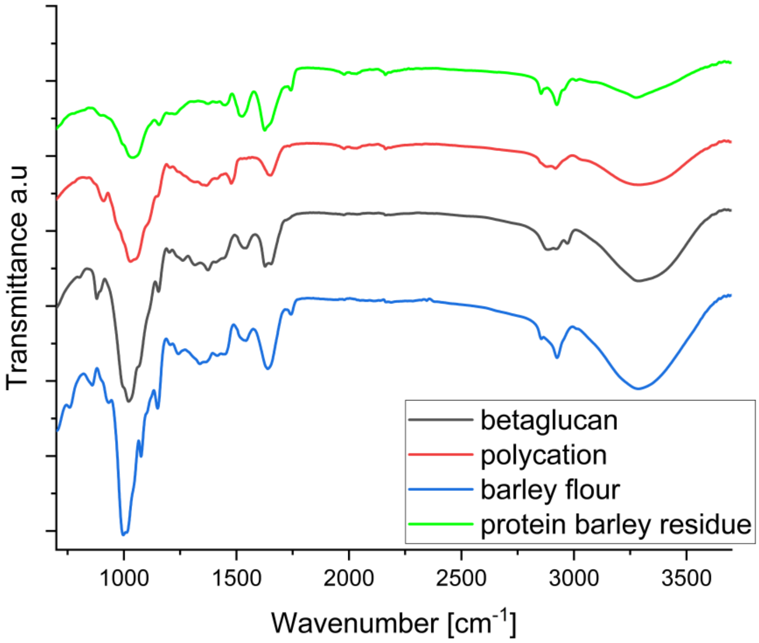

Figure S2. FTIR spectra of the products of successive steps in the isolation of beta-glucans from barley.

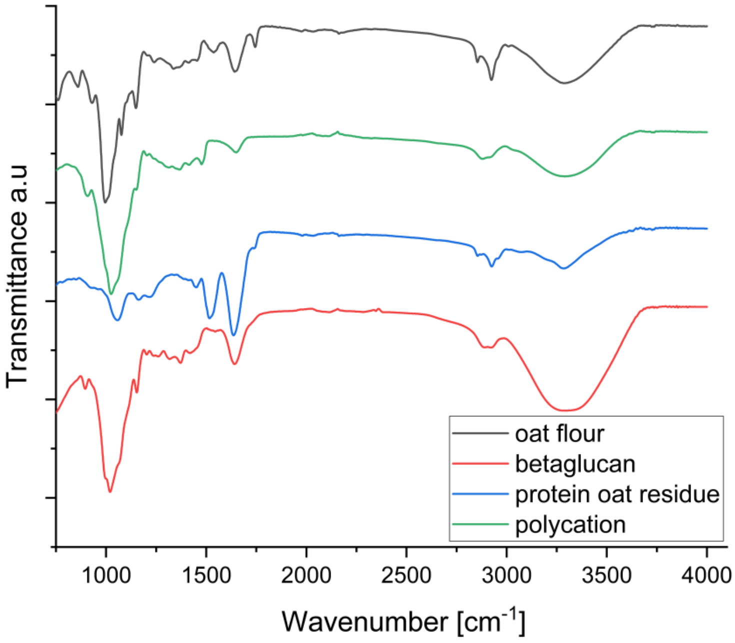

Figure S3. FTIR spectra of the products of successive steps in the isolation of beta-glucans from oat.

The IR spectra as well as the results from the elemental analysis confirm the fact of attachment of quaternary amines to the structures obtained, which is indicated by the peak at 1485cm^{-1}. The overall shape of the spectra obtained is characteristic of polysaccharides, especially beta-glucans. The spectra of cationic polymers obtained from beta-glucans originating from two sources are substantially similar. This may suggest that within the framework of the used isolation method the obtained compounds of this type will have a similar chemical structure regardless of the biological origin. To confirm these similarities, the two materials were examined using another spectroscopic method, which allows for deduction of the chemical structure, i.e. ^{1}H NMR.

1.3. ^{1}H NMR spectra

^{1}H NMR spectra of the two materials investigated were performed to confirm the similarity in chemical structure postulated in an earlier chapter. The beta-glucans extracted from oat and barley consists of a common structure, with primary backbone chains of (1/3) and (1/4)-linked -glucopyranosyl units along which are randomly dispersed-(1/6)-linked side chains. Collected ^{1}HNMR specra are very similar (see Figure S4). In each we can identify signals form glucose unit, as well as from cationic substituent; a singlet from H1 proton at 4.46 ppm, a multiplet in 4.12-3.36 ppm region from H2-H9 protons and a singlet at 3.21 ppm from methyl protons of of $–N(CH_3)_3$ group.

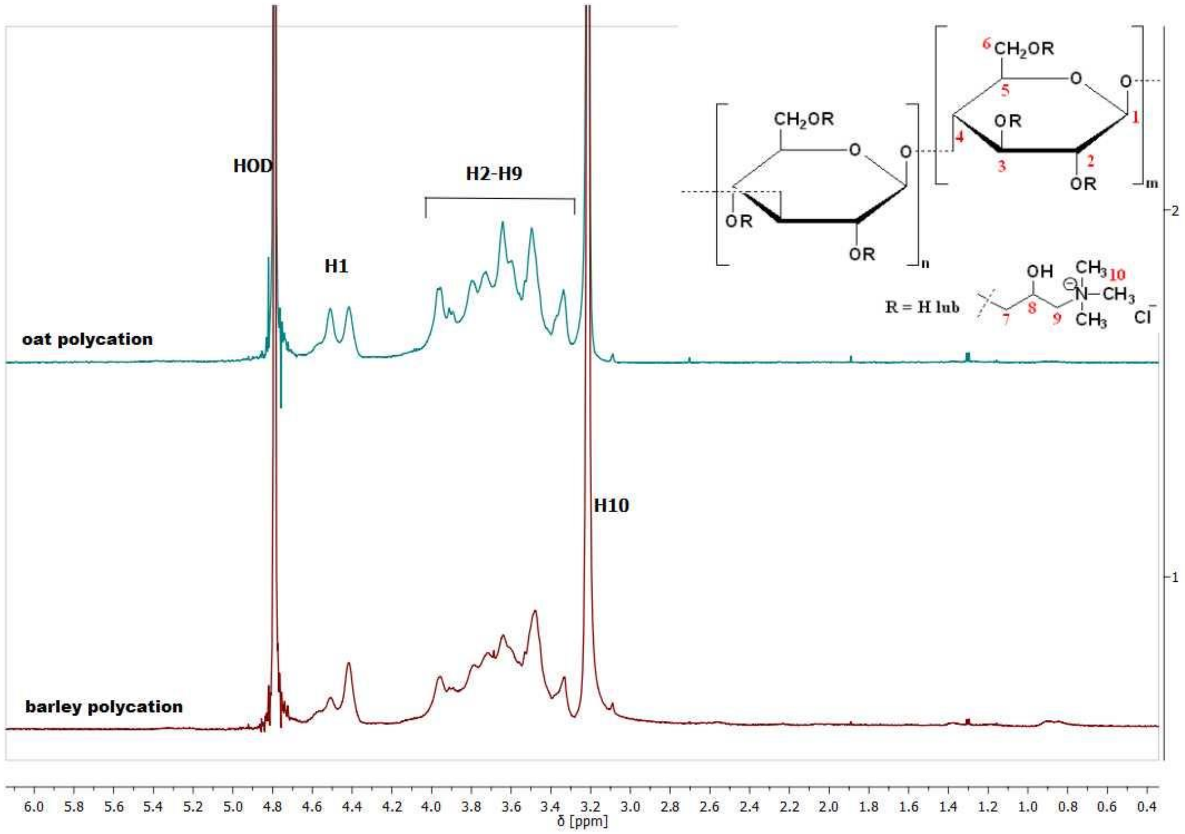

Figure S4. ^{1}H NMR spectras of oat and barley polycations in D_2O. In upper right corner: a potential structure of polycations obtained.

1.4. Determination of the degree of substitution

The degree of modification of the polysaccharide chain was determined using conductometric precipitation titration. Chloride ions, which are counter ions for the quaternary amine, were titrated using silver ions. The following relationship of conductivity to the amount of $AgNO_3$ added to the polycation solutions was obtained:

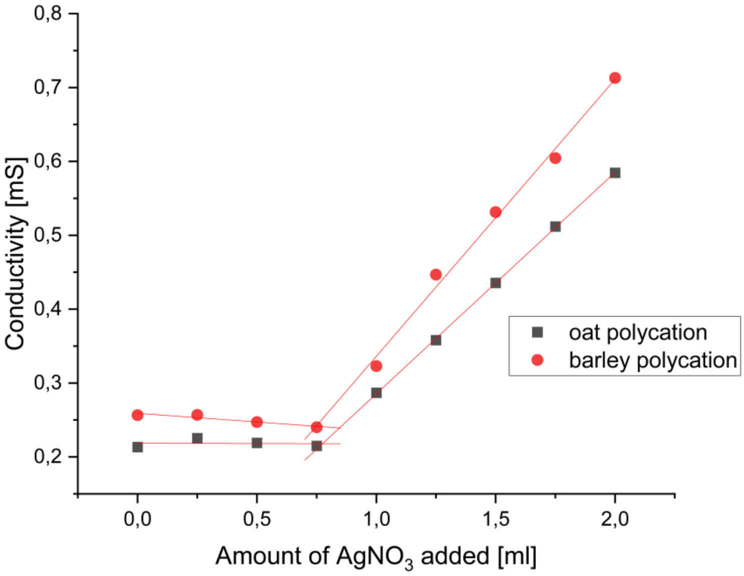

Figure S5. Dependence of conductivity [mS] on the amount of silver nitrate added in polycation precipitation titration.

Using the following equation [1], the degree of substitution (DS) of the two polycations was calculated:

$$DS\ [\%] = \frac{\frac{(1{,}7\times 10^{-3})V_{AgNO_3}}{W_{pk}-(1{,}7\times 10^{-3})V_{AgNO_3}m_{GTMAC}}}{m_g} \times 100 \qquad (1)$$

where W_{pk} is the mass of polycation in the sample (10 mg), m_{GTMAC} is the molar mass of GTMAC (151 g/mol), m_g is the molar mass of glucose (180 g/mol), V_{AgNO_3} is the volume of silver nitrate at the intersection of the two simple conductivity-volume relationships of silver nitrate (Figure S5).
